# Classifying healthcare facilities as predictors of COVID-19 mortality rates in US counties (2020–2021)

**DOI:** 10.1093/pubmed/fdag053

**Published:** 2026-06-26

**Authors:** Edwin M McCulley, Jana A Hirsch, Alina Schnake-Mahl, Brisa Sanchez, Gina S Lovasi, Usama Bilal

**Affiliations:** Department of Epidemiology & Biostatistics, Drexel University, 3215 Market Street, Philadelphia, PA 19104, USA; Urban Health Collaborative, Drexel University, 3215 Market Street, Philadelphia, PA 19104, USA; Department of Epidemiology & Biostatistics, Drexel University, 3215 Market Street, Philadelphia, PA 19104, USA; Urban Health Collaborative, Drexel University, 3215 Market Street, Philadelphia, PA 19104, USA; Urban Health Collaborative, Drexel University, 3215 Market Street, Philadelphia, PA 19104, USA; Department of Health Management & Policy, Drexel University, 3215 Market Street, Philadelphia, PA 19104, USA; Department of Epidemiology & Biostatistics, Drexel University, 3215 Market Street, Philadelphia, PA 19104, USA; Urban Health Collaborative, Drexel University, 3215 Market Street, Philadelphia, PA 19104, USA; Department of Epidemiology & Biostatistics, Drexel University, 3215 Market Street, Philadelphia, PA 19104, USA; Urban Health Collaborative, Drexel University, 3215 Market Street, Philadelphia, PA 19104, USA; Department of Epidemiology & Biostatistics, Drexel University, 3215 Market Street, Philadelphia, PA 19104, USA; Urban Health Collaborative, Drexel University, 3215 Market Street, Philadelphia, PA 19104, USA

**Keywords:** COVID-19, healthcare facilities, prediction, classification, mortality, healthcare accessibility

## Abstract

**Background:**

The COVID-19 pandemic disproportionately impacted vulnerable populations, with contextual factors like healthcare accessibility influencing mortality. However, limited evidence exists on which types of healthcare facilities affect COVID-19 death rates.

**Methods:**

We examined which facility types were statistically associated with, and improved prediction of, county-level COVID-19 mortality (2020–2021) using over dispersed Poisson models and healthcare facility data from the 2020 National Establishment Time Series database. Five feature selection strategies guided model construction: a theory-driven approach, three data-driven methods [Least Absolute Shrinkage and Selection Operator (LASSO), stepwise, and random forest], and a synthesized strategy integrating shared predictors.

**Results:**

Based on Quasi-Akaike’s Information Criterion (QAIC), LASSO and stepwise models offered the best fit. Across methods, consistent predictors of county-level COVID-19 mortality rates included pharmacies/drug stores, hospitals and major medical centers, emergency medical transport, offices and clinics of health practitioners, and urgent care facilities. Data-driven strategies also selected chiropractors, highlighting potential confounding bias.

**Conclusions:**

Our classification approach highlights facility types associated with COVID-19 mortality, offering insight into how healthcare infrastructure may influence pandemic-related health outcomes. These findings can support descriptive characterizations of local medical environments, generate hypotheses, and guide future research aimed at improving population health during public health emergencies.

## Background

The COVID-19 pandemic disproportionately affected vulnerable populations across the globe.^1^ In the USA, the pandemic highlighted racial/ethnic and spatial disparities exacerbating longstanding inequities like structural racism,^2–4^ and disparate socioeconomic status (SES).^5^ There have been investigations on the individual factors that predispose individuals to severe COVID-19 outcomes such as hospitalization and death,^6–8^ but fewer studies examine how local environments influence COVID-19 outcomes.^9^

One aspect that may influence COVID-19 outcomes is access to healthcare facilities. Research shows inequities in testing access, with socially vulnerable US cities having fewer testing sites compared to less vulnerable ones.^10^ Even when resources were available, many individuals delayed utilizing healthcare services.^11^^,^^12^ Staffing shortages and closures hindered access to care,^13^ including testing.^14^ While studies have assessed the role of healthcare resource availability,^13^^,^^15^^,^^16^ or access to healthcare services,^14^^,^^16^^,^^17^ there is a dearth of evidence on which types of healthcare facilities impact COVID-19 mortality.

Despite the evidence highlighting disparities in healthcare accessibility during the pandemic, many components of the medical environment remain understudied in relation to COVID-19 mortality. In this study, we review existing methods for classifying medical environment features,^18^^,^^19^ and propose several methods to determine which specific healthcare facilities improve model-based predictions of COVID-19 mortality in US counties, by constructing and comparing five feature selection strategies.

## Methods

### Study population & design

We utilized an ecological design to examine which types of healthcare facilities were statistically associated with COVID-19 mortality rates and improved predictive model performance. The study population included 3106 US counties eligible for linkage with exposure data on local healthcare facilities (37 excluded due to missing exposure data). We used 2020 bridged-race population estimates from the US Census Bureau.^20^

### Medical environment exposures

We obtained exposure data on healthcare facilities using the business establishment data for 2020 derived from the National Establishment Time Series (NETS) database,^21^ an annual census of US business, government, and non-profit establishments from Dun & Bradstreet.^19^ The dataset includes > 300 variables, like establishment name, address, industry classification, and relocation history, enabling time-series analyses.^21^ This data can be linked to neighborhood and health data to support research on environmental influences and health over time.^19^

We leveraged a healthcare facility classification scheme by Hirsch *et al.*,^19^ that hierarchically classifies different healthcare establishments. Each establishment was classified using a combination of Standard Industrial Classification codes and keyword or name searches, to identify “base groups” of facilities (e.g. urgent care). These base groups were combined to make larger “combination categories,” like community based care which includes retail clinics and urgent care. A description of each base group is provided in Table 1, and combination categories in the Supplementary Materials.

**Table 1:** Description of medical environment base groups in the 2020 NETS data.

**NETS** **base group**	**Description**	**Base group SIC codes** ^**a**^	**NETS** **combination category** ^**b**^
Behavioral health hospitals	Hospitals specializing in the treatment of substance abuse, alcoholism, and drug addiction (inpatient care) excluding residential homes for those with behavioral health problems. These locations provide acute care in emergency and in-patient care in serious conditions.	SIC codes 80690100, 80690101, 80690102	(i) Acute episodic care, (ii) All mental and behavioral health care, (iii) Hospital based inpatient care, (iv) All behavioral health care
Behavioral health outpatient and continuous care	Locations that provide longer-term, continuous, outpatient care including the treatment of substance abuse, alcoholism, and drug addiction.	SIC codes 80930100,80930101,80930102,80930103	(i) Ambulatory care, (ii) All mental and behavioral health care, (iii) All behavioral health care
Acupuncture and biofeedback	Acupuncture, naturopath, and biofeedback practitioners. May be relevant for older adults or aging.	SIC codes 80499901, 80499903, 80499908, 80939901	(i) Alternative medicine and other body work
Pediatric dental care	Providers (offices, clinics) that deliver dental services (i.e. dentists) required to maintain dental health specializing in pediatrics or child populations.	SIC code 80210106	None
Chiropractors	Chiropractors (alternate medicine and traditional medicine).	SIC code 80410000	(i) Ambulatory care, (ii) Alternative medicine and other body work
Children hospitals	Hospitals or major medical centers specializing in pediatrics or child populations.	SIC code 80699901	None
Offices or clinics of pediatric health practitioners	Offices and clinics of pediatric healthcare providers that are identified through SIC code as separate from hospitals and medical centers (may be co-located, but have their own SIC code)	SIC codes 80110516, 80420104	None
Dental care	Providers (offices, clinics) that deliver dental services (i.e. dentists) required to maintain dental health for adults. Does not include dentists specializing in pediatrics.	SIC codes in the range 80210100-80210105 or SIC Codes 80210000, 80210107, 80210108, 80210200, 80210201, 80210202, 80219902	(i) Ambulatory care
Pharmacies/drug stores—SIC code based definition	Drug stores and pharmacies. Locations representing stores where medicine and/or drugs are dispensed or sold. May not include those that are also supermarkets.	SIC code in the range 59120000-59129999 or SIC Code in 80110203	(i) All pharmacies/drug stores
Pharmacies/drug stores—chain name based definition	“The Drug Trade Channel includes stores that typically sell prescription pharmacy items and Health & BC products. Drug sub-channels include Rx Only & Small Independent Drug Stores and Conventional. A conventional drug store is a chain or independent retail store featuring prescription pharmacy items and/or HBC products. A Rx Only and Small Drug Store includes drug stores that derive less than 15% of their total revenue from the sale of items other than prescription drugs as well as other independent drug stores whose total annual volume is less than $1 million.” (from TD Retail Channel + Sub-Channel Definitions)	SIC codes 53000000-53999999, 54000000-54999999, 56000000-56999999, 59000000-59999999, 65120200-65120299 AND Company or Trade Name on the TDLinx list for trade channel “Drug” and sub-channels “Conventional Drug Store” or “Rx Only & Small Drug Store.”	(i) All pharmacies/drug stores
Hearing aids retail and testing locations	Locations to purchase and repair hearing aids and receive hearing testing. Relevant for older adults and aging.	SIC codes 50470306, 76290101, 80990202	None
Emergency medical transport	Emergency medical transport including ambulance services. While they may service a larger area than where they are location on the map, the density of them may be relevant for healthcare service times especially for older adult populations	SIC code 41199902	None
Hospitals and major medical centers	Hospitals or major medical centers for adults that are not specifically focused on behavioral or mental health. Does not include locations specializing in pediatrics.	SIC codes in the range 80620000-80629999 or SIC Codes 80110200, 80110201, 80690000, 80690200, 80690201, 80690300, 80690301, 80699900, 80699902, 80699903, 80699904, 80699905	(i) Hospital based inpatient care
Offices or clinics of health practitioners	Offices and clinics of healthcare providers for adults that are identified through SIC code as separate from hospitals and medical centers (may be co-located, but have their own SIC code). Does not include locations specializing in pediatrics.	SIC codes in the ranges 80110100-80110199, 80110500-80110514, 80119900-80119999, 80490100-80490199, 80930200-80930299, 80930300-80930399 or SIC codes 80110000, 80110202, 80110205, 80110517, 80110518, 80110519, 80110520, 80110522, 80110523, 80110524, 80310000, 80410000, 80420000, 80420100, 80420101, 80420102, 80420103, 80420105, 80429900, 80429901, 80430000, 80490000, 80499900, 80499902, 80499904, 80499906, 80499908, 80499909, 80930000, 80939900, 80939905, 80990200, 80999906, 80999907	(i) Ambulatory care
Kidney centers	Outpatient facilities that administer renal/kidney dialysis	SIC code 80920000	(i) Ambulatory care
Mental health hospitals	Hospitals specializing in the treatment of severe mental disorders (inpatient care) excluding residential homes for those with mental health problems. These locations provide acute care in emergency and serious conditions.	SIC codes 80630000, 80639900, 80639901	(i) Acute episodic care, (ii) All mental and behavioral health care, (iii) Hospital based inpatient care, (iv) All mental health care
Mental health outpatient and continuous care	Locations that provide continuous, outpatient care including both the prevention and treatment of mental disorders. These locations provide care for longer-term conditions of varying severity including low severity chronic mental health problems and ongoing treatment of serious conditions.	SIC codes 80110400, 80110401, 80110402, 80110403, 80490400, 80490401, 80490403, 80490404, 80939902	(i) Ambulatory care, (ii) All mental and behavioral health care, (iii) All mental health care
Optical	Locations to purchase and repair glasses and contact lenses and receive vision testing. May be relevant for older adults.	SIC codes in the ranges 50489901-50489903, 59950000-59959903	None
Physical and occupational therapy	Rehabilitative health services that use specially designed exercises and equipment to help individuals regain or improve their physical abilities.	SIC code 80110521, 80490200, 80490201, 80939903	(i) Ambulatory care, (ii) Alternative medicine and other body work, (iii) Physical therapy and massage
Residential facilities with health care	Residential facilities with health care. Includes nursing care facilities, convalescent homes, extended care facilities, nursing home, residential care for handicapped, geriatric residential care.	SIC codes in the range 83610400-83610499 or SIC codes 80510000, 80519900, 80519901, 80519902, 80520000, 80529900, 80529902, 80590000, 80599901, 80599904, 80599905, 80599906, 83610000, 83619900, 83619901, 83619904	None
Residential care	Residential care for services offered to old adults and those that require healthcare in their own home. This is not at a separate care facility, but instead an establishment that dispatches caretakers to homes. While the location of their work may not be the same as the business location, the density of this may signal amenities useful for aging.	SIC codes 80599902, 80820000, 80829900, 80829902	None
Retail clinics	Stand-alone (non-hospital) locations inside retail.	SIC code 80990103, 80990201	(i) Ambulatory care, (ii) Acute episodic care, (iii) Community based care
Urgent care	Stand-alone (non-hospital) locations able to treat acute medical problems. These places provide episodic care that is sporadic and discontinuous.	SIC code 80110204	(i) Ambulatory care, (ii) Acute episodic care, (iii) Community based care

a Adapted from Hirsch, J.A., Moore, K.A., Cahill, J., Quinn, J., Zhao, Y., Bayer, F.J., Rundle, A. and Lovasi, G.S., 2021. Business data categorization and refinement for application in longitudinal neighborhood health research: a methodology. *Journal of Urban Health*, *98*, pp. 271–284.

b Combination category definitions are available in Supplementary Materials, Supplementary Table S4. SIC, Standard Industrial Classification.

We aggregated the NETS census tract data to the county-level by summing the number of healthcare facilities present in each county. The primary exposure was operationalized as county-level per-capita spatial accessibility to specific types of healthcare facilities.^19^ For primary analyses, we utilized base groups of healthcare facilities as our exposure (except pharmacies/drug stores, for which the combination category was used), and utilized broader combination categories in our sensitivity analyses.

### COVID-19 mortality

Our primary outcome was crude county-level annual COVID-19 mortality rates among adults aged ≥18 years. We obtained 2020–2021 individual mortality records from the National Center for Health Statistics,^22^ geocoded to US counties. We identified all deaths attributable to COVID-19 using International Classification of Diseases 10th version codes U07.1 & U07.2, and aggregated data to provide annual COVID-19 mortality counts by county.^20^ To derive county-level mortality rates, the regression models employed included an offset for county population aged ≥18 years.

### Statistical analyses

We conducted this analysis in three steps: (i) we used 5 selection strategies to decide which types of healthcare facilities were predictive of county-level COVID-19 mortality rates, (ii) we fit Quasi-Poisson generalized linear models (QP GLMs) with selected features, a log-link function, a population offset, robust standard errors, and adjustment for percentage of county population aged ≥65 years, (iii) as we employed QP GLMs, traditional *R*^2^ values were not directly applicable, therefore we computed QAIC and compared the predictive performance of models. We intentionally did not adjust for additional county-level structural factors (e.g. SES, urbanicity) as the primary goal was predictive feature identification, rather than causal inference. In a predictive framework, the inclusion of covariates that are correlated with both the exposures and outcome can absorb shared variance and obscure the predictive signal of the features under evaluation, potentially undermining the feature selection process and complicating interpretation of which facility types contribute to predictive performance.^23^ Throughout this analysis, we use “predict” to refer to statistical associations improving model fit, not causality; therefore, “COVID-19 relevant” predictors describe features that improved predictive performance.

We first used a theory-driven strategy based on expert knowledge. Two authors screened the base groups to determine if each was “COVID-19 relevant,” based on the healthcare facilities involvement in the screening, treatment, or prevention of COVID-19 outcomes (i.e. infection, mortality); resulting in 95% agreement and one conflict, resolved via third party consensus. Second, we used stepwise backward variable selection.^24^ We began with a QP GLM that included all of the base groups as predictors, then iteratively identified and eliminated predictors with the highest Wald test statistic until all remaining predictors displayed a statistically significant (*P* < .05) association with COVID-19 mortality. Multicollinearity was not formally assessed within the stepwise approach; a recognized limitation, which we address by comparing results across multiple selection strategies, including Least Absolute Shrinkage and Selection Operator (LASSO) (which is more robust to collinearity via regularization) and the synthesized strategy (which requires convergence across strategies). Specific types of healthcare facilities were classified as “COVID-19 relevant” if they were included in the final model. Our third strategy used LASSO regularization. LASSO adds a penalty term (λ) that shrinks some coefficients to zero. We used ten-fold cross-validation to select optimal λ values minimizing test mean squared error; predictors with non-zero coefficients were classified as “COVID-19 relevant.” Fourth, we used the Boruta algorithm with a Random Forest model. The algorithm creates shadow features by randomly shuffling predictor values, trains a random forest model on the extended data, then computes and compares predictor importance scores against shadow feature scores. After 500 iterations, predictors with importance scores significantly exceeding random chance were classified as “COVID-19 relevant.” Prior to LASSO and random forest modeling, all predictors were standardized, mean-centered and scaled to unit variance, ensuring comparability across features. Our fifth strategy assumed no single best selection method,^25^ and leveraged a synthesized approach, which combined theory-driven and data-driven aspects, and selected “COVID-19 relevant” predictors if they were selected in a majority of the previous strategies. This consensus-based criterion aims to balance parsimony with robustness by identifying persistent features, without being overly permissive or restrictive, an approach aligned with recent literature.^26^^,^^27^

### Sensitivity analyses

We conducted two sensitivity analyses. First, we leveraged different exposures, the total number (count) of each type of healthcare facility present within a given county, and land area density (i.e. the number of facility type per square kilometer). Second, we repeated the primary analysis using the broader combination categories with additional analyses conducted for the theory-driven strategy, which classified combination categories as “COVID-19 relevant” if any one or more of the underlying base groups were initially classified as such using the theory-driven strategy.

## Results

The healthcare facilities selected as “COVID-19 relevant” predictors by each strategy are presented in Table 2. We found some overlap(s) between selection strategies, also noting differences. Our theory-driven strategy included 8 out of 22 base groups as COVID-19 relevant: all pharmacies/drug stores, emergency medical transport, hospitals and major medical centers, offices or clinics of health practitioners, residential facilities with health care, residential care, retail clinics, and urgent care. The stepwise and LASSO strategies showed a high degree of overlap, agreeing on 11 facility types: chiropractors, offices or clinics of pediatric health practitioners, dental care, all pharmacies/drug stores, hospitals and major medical centers, offices or clinics of health practitioners, kidney centers, mental health outpatient and continuous care, optical, physical and occupational therapy, and urgent care. The stepwise strategy further selected emergency medical transport (12 features total), while LASSO further selected acupuncture and biofeedback, pediatric dental care, and children hospitals (14 features total). The random forest strategy selected six healthcare facilities and showed overlap with the other strategies; agreeing on children hospitals with the LASSO strategy, residential facilities with health care in the theory-driven strategy, chiropractors with the stepwise and LASSO strategies, emergency medical transport with the theory-driven and stepwise strategies, plus agreement on all pharmacies/drug stores and hospitals and major medical centers with the theory-driven, stepwise, and LASSO strategies. Last, the synthesized strategy classified the following six base groups as COVID-19 relevant predictors: all pharmacies/drug stores, emergency medical transport, hospitals and major medical centers, offices or clinics of health practitioners, urgent care, and unexpectedly, chiropractors.

**Table 2:** Composition of candidate models for COVID-19 mortality and base groups per-capita.

**NETS base group**	**Theory**	**Stepwise**	**LASSO**	**Random forest**	**Synthesized**
Behavioral health hospitals					
Behavioral health outpatient and continuous care					
Acupuncture and biofeedback			✔		
Pediatric dental care			✔		
Chiropractors		✔	✔	✔	✔
Children hospitals			✔	✔	
Offices or clinics of pediatric health practitioners		✔	✔		
Dental care		✔	✔		
All pharmacies/drug stores	✔	✔	✔	✔	✔
Hearing aids retail and testing locations					
Emergency medical transport	✔	✔		✔	✔
Hospitals and major medical centers	✔	✔	✔	✔	✔
Offices or clinics of health practitioners	✔	✔	✔		✔
Kidney centers		✔	✔		
Mental health hospitals					
Mental health outpatient and continuous care		✔	✔		
Optical		✔	✔		
Physical and occupational therapy		✔	✔		
Residential facilities with health care	✔			✔	
Residential care	✔				
Retail clinics	✔				
Urgent care	✔	✔	✔		✔

We then compared the predictive performance of each model using QAIC (Table 3). Lower QAIC values suggest better model fit. In terms of base groups, the stepwise (QAIC_stepwise_ = 2453.9) and LASSO strategies (QAIC_LASSO_ = 2458.7) achieved the lowest QAIC; noting the stepwise result was expected given the algorithm directly optimizes this criterion. The synthesized (QAIC_synthesized_ = 2647.5) and theory-driven (QAIC_theory_ = 2699.2) models exhibited higher QAIC values but greater parsimony (6 and 8 features, respectively, versus 12 and 14). The random forest model (QAIC_randomforest_ = 2771.4) showed the lowest predictive performance; and all models outperformed the null model (QAIC_null_ = 3276.4).

**Table 3:** Predictive performance of candidate models (sorted from lowest to highest QAIC).

**Model**	**df** ^**a**^	**logLik**	**QAIC**	**ΔQAIC**
**Stepwise**	13	−51166.42	2453.9	0
**LASSO**	15	−51184.00	2458.7	4.83
**Synthesized**	7	−55506.68	2647.5	193.61
**Theory**	9	−56513.39	2699.2	245.30
**Random forest**	7	−58121.08	2771.4	317.46
**Null**	1	−69077.31	3276.4	822.49

a df denotes degrees of freedom.

Sensitivity analysis results are presented in the Supplementary Materials. Briefly, when using combination categories, ambulatory care, alternative medicine, community based care, and pharmacies/drug stores were consistently selected across the theory, stepwise, and LASSO strategies, with similar predictive performance. The random forest strategy again showed the lowest predictive performance. Consistent with primary analyses, operationalizing predictors as land area density or raw counts introduced variability in stepwise and LASSO results, with the random forest strategy becoming substantially less parsimonious.

## Discussion

### Main findings of this study

We aimed to compare selection strategies and classify healthcare facility types that are statistically associated with COVID-19 mortality in predictive models. This resulted in three key findings. First, while results varied across strategies, our synthesized strategy highlighted the salience of pharmacies/drug stores, hospitals and major medical centers, chiropractors, emergency medical transport, offices or clinics of health practitioners, and urgent care facilities, as predictors of COVID-19 mortality. Second, the stepwise and LASSO strategies achieved the best performance as measured by QAIC, while the synthesized and theory-based strategies traded predictive performance for greater parsimony and theoretical grounding. Third, sensitivity analysis results suggest that some methods were more sensitive to the operationalization of predictors, especially the random forest strategy.

The different types of healthcare facilities selected across models had roles relevant to COVID-19 mortality, except chiropractors. However, healthcare facility density is correlated with structural county characteristics (e.g. SES, urbanicity, racial/ethnic composition),^28^^,^^29^ and selected predictors may partly function as proxies for these factors. The interpretations below describe plausible mechanisms while acknowledging that structural confounding cannot be excluded (see Limitations). Pharmacies and drug stores were tasked with providing preventive services like testing and vaccinations.^30^ The role of offices or clinics of health practitioners during the pandemic differed from that of pharmacies. These facilities were not only responsible for providing preventive services, but also delivering routine care, and extending pandemic-related services.^31^^,^^32^ This is important considering chronic diseases, like cardiovascular disease which increase the risk of severe COVID-19,^6^^,^^33–36^ and fluctuations in health care utilization which may have led to foregone medical care.^12^^,^^37^ The extension of health services offered by offices or clinics of health practitioners was also observed among urgent care clinics which expanded telemedicine services and served as points of routine and preventive care,^38^^,^^39^ notwithstanding changing patterns of health care utilization.^40^ Hospitals and major medical centers also played a crucial role during the pandemic, as these facilities were responsible for providing routine, emergency and specialized services, like intensive care units and surgical suites.^41^ Moreover, hospitals provided durable medical equipment, like ventilators and operating room machinery^42^; which were critical to pandemic response. Like hospitals, emergency medical transport was also important to pandemic-related care by providing emergency services and transporting patients.^43^^,^^44^

Differences in model composition resulted in differences in predictive performance. As noted, the stepwise strategy’s lowest QAIC reflects algorithmic optimization of this criterion. However, stepwise selection is susceptible to overfitting, multicollinearity, and model instability,^45^ though the high overlap with LASSO results (11 shared features) provides indirect evidence that multicollinearity did not substantially distort selection. LASSO yielded similar performance with greater robustness to overfitting via cross-validation, though it remains sensitive to strong multicollinearity and λ misspecification.^46^^,^^47^ The random forest strategy exhibited lower predictive performance, potentially attributable to variability introduced by random shuffling of shadow features across iterations.^48^ While the data-driven strategies generally achieved lower QAIC values, the synthesized strategy is not intended as an optimal predictive solution but rather as a practical compromise that balances statistical fit with parsimony and theoretical coherence. Its value lies in identifying a concise, consistently selected, and interpretable set of predictors; qualities not captured by QAIC alone.

### What is already known on this topic?

Epidemiology has historically trended towards preferring theory-driven selection as opposed to data-driven strategies,^25^^,^^49^ but recent work suggests opportunities for enhancing theory with machine learning and other data-driven strategies^49^^,^^50^; like mixture models which are common to environmental epidemiology and recently adapted to social epidemiology.^51^ Strictly relying on data-driven selection is limited by the absence of theory, high dimensional correlated exposures, and the prioritization of flexibility methods that are difficult to interpret over restrictive methods that afford more interpretable results.^51^ In light of these trends and the limitations of data-driven strategies, theory-driven tools, like a priori hypotheses and directed acyclic graphs, can be used to incorporate a priori subject matter knowledge pertinent to feature selection.^49^

### What this study adds

Our approach highlights the salience of pharmacies/drug stores, emergency medical transport, offices or clinics of health practitioners, urgent care, and hospitals/major medical centers, as relevant predictors of COVID-19 mortality in US counties. The consistent selection of chiropractors across data-driven strategies represents an intriguing finding warranting further investigation. While we hypothesize this reflects unmeasured confounding given established associations between chiropractor utilization,^52^ socioeconomic status,^53^^,^^54^ and urbanicity,^53^^,^^55^ alternative explanations including health insurance coverage,^56^^,^^57^ and proxies for other health-seeking behaviors or structural factors, cannot be ruled out. The chiropractor finding exemplifies a broader challenge in this study; in the absence of adjustment for structural covariates, it is difficult to disentangle whether selected facility types predict COVID-19 mortality because of their direct role in healthcare delivery, or because their geographic distribution reflects underlying county-level characteristics that independently influence mortality risk. Disambiguating these pathways is therefore a priority for future work.

Our synthesized strategy can identify medical environment features that improve prediction of COVID-19 mortality and guide future research aimed at reducing inequities, especially among vulnerable populations. Pending external validation, these findings could support surveillance, characterize county-level variation in facility distribution and generate hypotheses, warranting additional investigation before informing policy or interventions. The theory-driven strategy’s lower predictive performance likely reflects its causal orientation; prediction tasks benefit from algorithmic optimization, while causal tasks require a priori knowledge.^58^ The theory and synthesized strategies differed by only one feature (chiropractors), underscoring theory’s role in constraining data-driven selection. In future research, we will test the synthesized strategy with a causal goal in mind, as this strategy encompasses aspects of both data- and theory-driven strategies.

### Limitations of the study

In terms of limitations, the absence of county-level structural covariates (e.g. SES, urbanicity) is an important constraint. As previously noted, this was deliberate given our predictive goal; however, selected predictors may function as proxies for broader contextual factors across all facility types. Future analyses incorporating structural adjustment are needed to determine whether these associations persist after accounting for contextual factors. As this is an ecological analysis, the associations observed at the county-level may not reflect individual-level risk, a limitation known as the ecological fallacy. Accordingly, our findings should not be interpreted as evidence that redistributing specific facility types would reduce COVID-19 mortality; rather, they support hypothesis generation and planning for future work. The potential for information bias is another limitation; COVID-19 deaths may have been misclassified on death certificates,^59^ which was widespread early in the pandemic.^60^ Additionally, while LASSO incorporated internal cross-validation, we did not perform external validation (e.g. split-sample or temporal validation), as our primary aim was feature identification rather than model deployment; internal cross-validation was deemed appropriate for this purpose. However, generalizability to other populations, time periods, or geographic contexts remains uncertain, and external validation is recommended before practical application. Finally, our measure of accessibility, is time-fixed which ignores the closure/opening of healthcare facilities in 2021 and fails to capture differences in catchment areas. For example, hospitals serve more patients than doctor’s offices. Future research with time-varying facility and catchment area data could provide more precise estimates of these relationships.

In terms of strengths, we used five feature selection strategies, including data-driven strategies that leverage cross-validation to avoid overfitting, a theory-driven strategy that recognizes the importance of theory, and a synthesized strategy that harnesses benefits from both types of strategies, which aligns with literature on defining a limited set of exposure mixtures in social epidemiology.^51^ Finally, given our focus on COVID-19 mortality, our synthesized strategy may be adapted to additional outcomes (especially COVID-19 outcomes), as it can inform the selection of complex, multifactorial place-based exposures. An important next step is to re-evaluate the features identified herein using a causal framework that incorporates parsimonious structural adjustment to assess whether the predictive associations observed reflect healthcare access mechanisms, structural confounding, or both.

## Conclusion

This work provides an empirical basis for identifying healthcare facility types associated with pandemic-related mortality, which can support the characterization of healthcare environments, and upon further validation, may help generate hypotheses and inform research in the face of future public health emergencies.

## Supplementary Material

Supplement_v25_clean_fdag053

## Data Availability

The data that support the findings of this study are available from multiple sources. County-level mortality data are publicly available from the National Center for Health Statistics (NCHS). Medical environment data were obtained from the National Establishment Time-Series (NETS) database under a licensing agreement and are not publicly available. Restrictions apply to the availability of these data, which were used under license for the current study. Derived analytic data and code may be made available from the corresponding author upon reasonable request, subject to applicable data use agreements and licensing restrictions.

## Notes

**Edwin M. McCulley**, PhD, MS, Doctoral Research Fellow

**Jana A. Hirsch**, PhD, MES, Associate Research Professor

**Alina Schnake-Mahl**, ScD, MPH, Assistant Professor

**Brisa N. Sánchez**, PhD, Dornsife Endowed Professor of Biostatistics and Associate Dean for Research

**Gina S. Lovasi**, PhD, MPH, Dana and David Dornsife Dean of the Dornsife School of Public Health and Professor of Epidemiology

**Usama Bilal**, MD, PhD, MPH, Co-Director of the Urban Health Collaborative and Associate Professor
