# Supplementary material for: Classifying healthcare facilities as predictors of COVID-19 mortality rates in US counties (2020–2021)
Source: J Public Health (Oxf). 2026 Jun 26;48(3):680–90. doi: 10.1093/pubmed/fdag053 (PMC13531722; doi:10.1093/pubmed/fdag053)
Supplement: Supplementary file 1 [file supplement_v25_clean_fdag053.docx]

**Table S1) Composition of Candidate Models for COVID-19 Mortality and Base Groups: Per-capita, Land Area and Count**

| **NETS**  **Base Group** | **Abbrev.** | **Theory** | | | **Stepwise** | | | **LASSO** | | | **Random Forest** | | | **Synthesized** | | |
| --- | --- | --- | --- | --- | --- | --- | --- | --- | --- | --- | --- | --- | --- | --- | --- | --- |
|  |  | **Per Capita** | **Land Area** | **Count** | **Per Capita** | **Land Area** | **Count** | **Per Capita** | **Land Area** | **Count** | **Per Capita** | **Land Area** | **Count** | **Per Capita** | **Land Area** | **Count** |
| **Behavioral Health Hospitals** | BHH |  |  |  |  |  |  |  |  |  |  |  | ✔ |  |  |  |
| **Behavioral Health Outpatient and Continuous Care** | BHO |  |  |  |  |  | ✔ |  |  | ✔ |  | ✔ | ✔ |  |  | ✔ |
| **Acupuncture and Biofeedback** | BIO |  |  |  |  |  |  | ✔ |  |  |  | ✔ | ✔ |  |  |  |
| **Pediatric Dental Care** | CDS |  |  |  |  | ✔ | ✔ | ✔ |  | ✔ |  |  | ✔ |  |  | ✔ |
| **Chiropractors** | CHR |  |  |  | ✔ | ✔ | ✔ | ✔ |  | ✔ | ✔ | ✔ | ✔ | ✔ |  | ✔ |
| **Children Hospitals** | CHS |  |  |  |  | ✔ | ✔ | ✔ |  |  | ✔ |  |  |  |  |  |
| **Offices or clinics of pediatric health practitioners** | CPC |  |  |  | ✔ | ✔ | ✔ | ✔ |  | ✔ |  | ✔ | ✔ |  |  | ✔ |
| **Dental Care** | DDS |  |  |  | ✔ |  | ✔ | ✔ |  | ✔ |  |  | ✔ |  |  | ✔ |
| **All Pharmacies/Drug Stores** | DRA | ✔ | ✔ | ✔ | ✔ | ✔ | ✔ | ✔ | ✔ | ✔ | ✔ | ✔ | ✔ | ✔ | ✔ | ✔ |
| **Hearing aids retail and testing locations** | EAR |  |  |  |  | ✔ | ✔ |  |  |  |  | ✔ |  |  |  |  |
| **Emergency medical transport** | EMT | ✔ | ✔ | ✔ | ✔ | ✔ | ✔ |  | ✔ | ✔ | ✔ | ✔ | ✔ | ✔ | ✔ | ✔ |
| **Hospitals and major medical centers** | HOS | ✔ | ✔ | ✔ | ✔ | ✔ |  | ✔ |  |  | ✔ | ✔ | ✔ | ✔ | ✔ |  |
| **Offices or clinics of health practitioners** | HPC | ✔ | ✔ | ✔ | ✔ |  | ✔ | ✔ |  |  |  | ✔ | ✔ | ✔ |  | ✔ |
| **Kidney Centers** | KCT |  |  |  | ✔ |  |  | ✔ |  |  |  | ✔ | ✔ |  |  |  |
| **Mental Health Hospitals** | MHH |  |  |  |  | ✔ |  |  |  |  |  |  | ✔ |  |  |  |
| **Mental Health Outpatient and Continuous Care** | MHO |  |  |  | ✔ | ✔ |  | ✔ |  |  |  | ✔ | ✔ |  |  |  |
| **Optical** | OPT |  |  |  | ✔ |  | ✔ | ✔ |  |  |  | ✔ | ✔ |  |  |  |
| **Physical and Occupational Therapy** | PHT |  |  |  | ✔ | ✔ | ✔ | ✔ |  |  |  | ✔ | ✔ |  |  |  |
| **Residential facilities with health care** | RES | ✔ | ✔ | ✔ |  |  |  |  |  | ✔ | ✔ | ✔ | ✔ |  |  | ✔ |
| **Residential Care** | RSC | ✔ | ✔ | ✔ |  | ✔ |  |  |  |  |  | ✔ | ✔ |  | ✔ |  |
| **Retail clinics** | RTC | ✔ | ✔ | ✔ |  |  |  |  |  |  |  | ✔ | ✔ |  |  |  |
| **Urgent care** | URG | ✔ | ✔ | ✔ | ✔ | ✔ | ✔ | ✔ |  | ✔ |  | ✔ |  | ✔ | ✔ | ✔ |
| Note: ✔ denotes inclusion in candidate model | | | | | | | | | | | | | | | | |

**Table S2) Predictive Performance of Candidate Models for Base Groups: Land Area**

| **Model** | **df** | **logLik** | **QAIC** | **ΔQAIC** |
| --- | --- | --- | --- | --- |
| **Stepwise** | 15 | -55902.95 | 2680.3 | 0 |
| **Random Forest** | 20 | -58018.89 | 2790.5 | 110.24 |
| **Theory** | 10 | -61825.88 | 2950.9 | 270.59 |
| **Synthesized** | 7 | -62212.95 | 2963.2 | 282.92 |
| **LASSO** | 4 | -64376.97 | 3059.7 | 379.44 |
| **Null** | 1 | -69077.31 | 3276.4 | 596.11 |

**Table S3) Predictive Performance of Candidate Models for Base Groups: Count**

| **Model** | **df** | **logLik** | **QAIC** | **ΔQAIC** |
| --- | --- | --- | --- | --- |
| **Stepwise** | 15 | -55000.75 | 2637.5 | 0 |
| **Synthesized** | 12 | -56644.36 | 2709.4 | 71.86 |
| **LASSO** | 11 | -56970.03 | 2722.8 | 85.29 |
| **Random Forest** | 21 | -57896.73 | 2786.7 | 149.19 |
| **Theory** | 10 | -58628.94 | 2799.4 | 161.88 |
| **Null** | 1 | -69077.31 | 3276.4 | 638.85 |

**Table S4) Medical Environment Features – Combination Categories**

| **NETS Combination Category** | **Abbrev.** | **Combination Category Description & Constituent Base Groups** |
| --- | --- | --- |
|  |  |  |
| **Acute Episodic Care** | AEC | Locations able to treat acute medical problems for adults. These places provide episodic care that may be sporadic and discontinuous. They do not provide chronic, continuous care. Does not include locations that specialize in pediatrics. *Includes BHH, MHH, RTC, or URG.* |
| **Ambulatory Care** | AMB | Locations able to treat and provide chronic, continuous, outpatient care including screenings and other preventive measures for adults. These places both allow an individual to maintain their health and address non-acute, non-emergency conditions. Does not include locations specializing in pediatrics. *Includes BHO, CHR, DDS, HPC, KCT, MHO, PHT, RTC, or URG.* |
| **Alternative medicine and other body work** | ATR | Alternative medicine and other body work with the goal of healing (including massage, acupuncture, biofeedback, etc). *Includes BIO, CHR, or PHT.* |
| **All Behavioral Health Care** | BHA | Hospitals and continuous, outpatient care specializing in the treatment of substance abuse, alcoholism and drug addiction. *Includes BHH and BHO.* |
| **Community Based Care** | CMB | Stand-alone (non-hospital) locations able to treat acute medical problems including those inside retail for adults. These places provide episodic care that is sporadic and discontinuous. Does not include locations specializing in pediatrics. *Includes RTC or URG.* |
| **All Pharmacies/Drug Stores** | DRA | Drug stores and pharmacies. Locations representing stores where medicine and/or drugs are dispensed or sold. May not include those that are also supermarkets. Includes pharmacies and drug stores defined by SIC code OR having a chain name on the TDLinx name list. *Includes DRG or DRN.* |
| **Hospital based inpatient care** | HOI | All hospitals or major medical centers for adults. Does not include locations specializing in pediatrics. *Includes BHH, HOS, or MHH.* |
| **All Mental and Behavioral Health Care** | MBH | Locations to aid in the prevention and treatment of mental and behavioral health issues, including depression and drug abuse. This category excludes long-term residential locations and pediatric-specific facilities. *Includes BHH, BHO, MHH, or MHO.* |
| **All Mental Health Care** | MHA | Hospitals and continuous, outpatient care specializing in the treatment of mental disorders. Does not include locations specializing in pediatrics. *Includes MHH or MHO.* |
| **Physical Therapy and Massage** | PTM | This category combines physical therapists with massage parlors. Massage parlors can be distinguished from the similar “spa” in that they mainly do sports medicine massage, etc. This overall category includes the formal, medical “physical therapist” with the less formal “massage parlor.” *Includes PHT and massage parlors (MAS), which are not included as features of the medical environment.* |

**Table S5) Composition of Candidate Models for COVID-19 mortality and Combination Categories: Per-capita, Land Area and Count**

| **NETS Combination Category** | **Abbrev.** | **Theory** | | | **Stepwise** | | | **LASSO** | | | **Random Forest** | | | **Synthesized** | | |
| --- | --- | --- | --- | --- | --- | --- | --- | --- | --- | --- | --- | --- | --- | --- | --- | --- |
|  |  | **Per Capita** | **Land Area** | **Count** | **Per Capita** | **Land Area** | **Count** | **Per Capita** | **Land Area** | **Count** | **Per Capita** | **Land Area** | **Count** | **Per Capita** | **Land Area** | **Count** |
| **Acute Episodic Care** | AEC | ✔ | ✔ | ✔ | ✔ | ✔ | ✔ |  |  | ✔ | ✔ | ✔ | ✔ | ✔ | ✔ | ✔ |
| **Ambulatory Care** | AMB | ✔ | ✔ | ✔ | ✔ | ✔ | ✔ | ✔ |  | ✔ |  | ✔ | ✔ | ✔ | ✔ | ✔ |
| **Alternative medicine and other body work** | ATR | ✔ | ✔ | ✔ | ✔ | ✔ | ✔ | ✔ | ✔ | ✔ |  | ✔ | ✔ | ✔ | ✔ | ✔ |
| **All Behavioral Health Care** | BHA |  |  |  |  |  |  |  |  | ✔ | ✔ | ✔ | ✔ |  |  |  |
| **Community Based Care** | CMB | ✔ | ✔ | ✔ | ✔ | ✔ | ✔ | ✔ | ✔ | ✔ |  | ✔ | ✔ | ✔ | ✔ | ✔ |
| **All Pharmacies/Drug Stores** | DRA | ✔ | ✔ | ✔ | ✔ |  | ✔ | ✔ | ✔ | ✔ | ✔ | ✔ | ✔ | ✔ | ✔ | ✔ |
| **Hospital based inpatient care** | HOI | ✔ | ✔ | ✔ |  |  |  | ✔ |  | ✔ | ✔ | ✔ | ✔ | ✔ |  |  |
| **All Mental and Behavioral Health Care** | MBH |  |  |  | ✔ | ✔ | ✔ | ✔ |  | ✔ | ✔ | ✔ | ✔ | ✔ |  | ✔ |
| **All Mental Health Care** | MHA |  |  |  |  |  |  | ✔ | ✔ | ✔ | ✔ | ✔ | ✔ |  |  |  |
| **Physical Therapy and Massage** | PTM |  |  |  |  |  | ✔ |  |  | ✔ |  | ✔ | ✔ |  |  | ✔ |
| Note: ✔ denotes inclusion in candidate model | | | | | | | | | | | | | | | | |

**Table S6) Predictive Performance of Candidate Models for Combination Categories: Per-capita**

| **Model** | **df*** | **logLik** | **QAIC** | **ΔQAIC** |
| --- | --- | --- | --- | --- |
| **Stepwise** | 8 | -53168.9 | 2536.755 | 0 |
| **Synthesized** | 8 | -53168.9 | 2536.755 | 0 |
| **LASSO** | 9 | -53168.7 | 2538.747 | 1.992 |
| **Theory** | 7 | -54416.8 | 2593.873 | 57.118 |
| **Random Forest** | 7 | -58492.4 | 2786.944 | 250.189 |
| **Null** | 1 | -69077.3 | 3276.38 | 739.625 |

**Note:** *****df denotes degrees of freedom

**Table S7) Predictive Performance of Candidate Models for Combination Categories: Land Area**

| **Model** | **df*** | **logLik** | **QAIC** | **ΔQAIC** |
| --- | --- | --- | --- | --- |
| **Stepwise** | 7 | -60195.9 | 2867.644 | 0 |
| **Random Forest** | 10 | -60162.4 | 2872.055 | 4.411 |
| **Theory** | 7 | -62020.1 | 2954.061 | 86.417 |
| **Synthesized** | 7 | -62020.1 | 2954.061 | 86.417 |
| **LASSO** | 6 | -62738.6 | 2986.098 | 118.454 |
| **Null** | 1 | -69077.3 | 3276.38 | 408.736 |

**Note:** *****df denotes degrees of freedom

**Table S8) Predictive Performance of Candidate Models for Combination Categories: Count**

| **Model** | **df*** | **logLik** | **QAIC** | **ΔQAIC** |
| --- | --- | --- | --- | --- |
| **Stepwise** | 9 | -60090.1 | 2866.633 | 0 |
| **Synthesized** | 9 | -60090.1 | 2866.633 | 0 |
| **LASSO** | 10 | -60059.1 | 2867.161 | 0.528 |
| **Random Forest** | 10 | -60059.1 | 2867.161 | 0.528 |
| **Theory** | 7 | -62132.2 | 2959.37 | 92.737 |
| **Null** | 1 | -69077.3 | 3276.38 | 409.747 |

**Note:** *****df denotes degrees of freedom
